# The Family System of Sexuality Communication: Extended Family Perceptions of Adolescent–Family Talk about Sex, with Sibling and Non-Sibling Comparison

**DOI:** 10.3390/sexes2010001

**Published:** 2020-12-30

**Authors:** Jennifer M. Grossman, Nora Pearce, Amanda M. Richer

**Affiliations:** Wellesley Centers for Women, Wellesley College, 106 Central Street, Wellesley, MA 02481, USA;

**Keywords:** adolescent health, extended family, family communication, sexual health, sexuality communication, sibling

## Abstract

Talk with parents and extended family about sex and relationships can support adolescents’ sexual health. However, few studies explore how parent and extended family communication with adolescents intersect. This study used thematic analysis to assess family roles in talk with teens about sex and relationships among a sample of 39 adult extended family members (such as aunts and uncles, and older siblings and cousins) in the United States. Analyses identified four themes in sexuality communication that address: why adolescents talk to extended family about sex and relationships, family engagement in these conversations, consistency of family messages, and family communication about adolescents. Findings identify variation in how family members interact with adolescents and one another regarding talk about sex and relationships. For example, some participants described family coordination of sexual messages to the teen, while others reported no family communication about this topic. Results also showed similarities and differences in how sibling and non-sibling extended family describe these processes. These findings identify the need to examine family talk about sex and relationships in the context of a larger family system, rather than only within dyadic relationships, and suggests possibilities for family-based interventions to support adolescents’ sexual health.

## 1. Introduction

A large body of research shows that parent–teen talk about sex is associated with delayed sex [1] and reduced adolescent sexual risk behavior [2]. However, half of adolescents report that they do not talk with parents about sex [3]. This in part reflects adolescents’ feelings of discomfort and awkwardness in talking with parents about sexual issues and their worries that parents will judge them or be disappointed in them due to their sexual behavior [4,5]. Despite parent–teen sexuality communication limitations, most research focuses on a parent–teen dyad, specifically the mother–teen dyad [2,6]. While mothers play a key role in teens’ family sexuality communication [7], adolescents’ communication about sex goes beyond mothers to include a larger family network [8–10]. Therefore, it is important to understand how other family members can take on the role of talking with teens about sex, either supplementing parent–teen conversations about sex, or filling in when parents and teens do not have these conversations. However, the larger family system of sexuality communication has rarely been explored.

Extended family members, including non-parental family such as aunts and uncles, cousins, siblings, and grandparents [11,12] can play an important role in talking with adolescents about sex and relationships [8,9]. Studies show that close to half of high school-aged teens report talking with an extended family member about sex or relationships [11]. Further, 41% of adolescents report talk with *both* parents and extended family about sex and relationships [9], which shows the need to understand family communication as a network rather than a dyad. These findings suggest that extended family, in addition to parents, serve as sources of sexual socialization for adolescents, providing a means for families to pass on their sexual values and beliefs [13].

Recent quantitative research suggests that talk with extended family can support teens’ sexual health, with findings that extended family talk with teens about protection was associated with fewer sexual partners [11]. Qualitative findings suggest that teens often feel comfortable talking with extended family members about sex in part due to shared life experiences [5]. A study of parent and grandparent caregivers found that grandparents were more open to and interested than parents in talking with their adolescent grandchildren about sex [8]. Parents and extended family may also discuss different topics with adolescents, with parents more likely than extended family to focus on delaying sex [5,14]. Extended family often talk with adolescents about topics such as safer sex, sexually transmitted infections (STIs), and sexual orientation [12], which parents may be hesitant to address [15,16]. These findings suggest that parents and extended family may play different roles in talking with adolescents in their families about sex and relationships.

While prior research has explored similarities and differences in the content of adolescents’ talk with parents and extended family [5], few studies assess how parents and extended family members interact with adolescents and one another regarding talk about sex and relationships. One example is a qualitative study, which found that parents often described bringing in extended family members as supports for talk with their adolescents about sex [17]. Extending research beyond the parent–teen dyad to explore the family system of sexuality communication can help us understand how adolescents’ larger family networks interact to shape teens’ sexual health. However, to our knowledge, no studies focus on how extended family, parents, and adolescents interact and shape the adolescents’ family systems of talk about sex. In addition, with few exceptions, e.g., [8,12], little research includes extended family perspectives on family talk about sex.

Few studies have explored family systems of sexuality communication, but research stemming from family systems theory [18] has identified the importance of investigating the larger family system to understand children’s development, rather than assessing parent–child relationships in isolation [19]. Research suggests that how family systems interact can impact youth development [20,21]. For example, research has explored how sibling relationships interact with parent–child relationships, e.g., [22]. Some approaches view sibling relationships as modeled based on parent–teen relationships, identified as congruence [23], while compensation approaches propose that other family relationships (such as siblings or other non-parental relatives) can make up for negative or absent parental relationships [24]. This theoretical and empirical work suggests the need to understand the roles parents and other family members play in talk with teens about sex and relationships.

Family talk about sex is shaped by a number of factors, such as gender, racial/ethnic background, religiosity, and sexual orientation [2,25,26]. For example, studies show that the content of parents’ talk with teens about sex as well as the effects of these conversations on teens’ sexual behavior, varies based on teens’ gender [2,27,28]. Initial research suggests that the content of extended family talk with teens about sex may be more similar across teen gender as compared to parents [29]. Racial and ethnic background may also shape family sexuality communication in multiple ways. For instance, cultural (and religious) taboos about sexual behavior and conversations about sex can inhibit talk about sex in Latino families [27]. On the other hand, the high cultural value attributed to extended-family involvement in many Black and Latino families [25,30] may provide opportunities for teens to talk with extended family about sex. Teens’ sexual orientation also shapes whether and how parents and extended family talk with teens about sex. Parents of sexual minority teens may avoid talking with their teens about sex due to discomfort or lack of knowledge on how to address sexual minority issues [26,31]. However, extended family may be a resource in this area, with high levels of talk with teens about sexual orientation [12]. It is also important to keep in mind the variety of living configurations for teens and their families, as 20% of families live with multiple generations in one household, and is particularly high for Asian and Latino families [32]. Whether the teen lives with extended family may help to understand family communication networks for talk about sex and relationships. These aspects of families’ and adolescents’ backgrounds and identities help shape whether and how families communicate about sex and relationships.

Siblings may play a unique role in teens’ sexuality communication, as the most common source of non-parental talk with teens about sex [11,33]. A study of family sexuality communication found that older sisters often play mentoring roles for teens in areas of romantic relationships and sexuality [34]. We conceptualize siblings as part of the teens’ extended family due to studies which suggest commonalities between siblings’ and cousins’ talk with teens about sex and relationships [10,35]. However, while the content of their talk with teens has much in common with other extended family members, a qualitative study found that siblings may be more likely to talk about sexual orientation, whereas other extended family may be more likely to talk with teens about protection methods, teen pregnancy, and misinformation about sex [12]. Research also suggests a potential protective role for siblings against adolescent sexual risk behavior, e.g., [33]. A better understanding of sibling and non-sibling extended family roles can help differentiate how different family members may contribute to talk with teens about sex and relationships.

The current study explores extended family members’ perspectives on how parent and extended family sexuality communication intersect and whether and how family members communicate with one another about adolescents’ sexual health. In addition, it includes an initial comparison of how siblings describe talk with adolescents and other family members about sex and relationships, compared to reports of non-sibling participants.

## 2. Materials and Methods

### 2.1. Recruitment and Participants

This study is based on interviews conducted with 39 extended family members living in the US who reported that they talk to a high school-aged adolescent in their families about sex and relationships. All 39 participants gave their informed consent before they participated in the study. Participants were informed about the purpose of the study, the costs and benefits of participation, that participation was voluntary, and that they could stop the interview at any time, and they were assured that their privacy would be protected. Each participant was offered a $25 gift card to Amazon, CVS pharmacy, or Target as appreciation for their participation. This study’s protocol was approved by the Wellesley College Institutional Review Board (19 December 2016) and adhered to the Declaration of Helsinki. Participants were recruited through local schools and organizations, social media platforms like Facebook, and national sites like Amazon Mechanical Turk (MTurk). Before conducting the interviews, interested parties took a screening survey to answer three questions: (1) Do you have a younger sibling, cousin, niece or nephew or grandchild in high school? (2) Do you talk with them about dating, sex, or relationships? (3) Are you 18 or older? If they answered “yes” to all three survey questions, these individuals were given consent information and invited to partake in the study.

Most participants (46%) were 20–25 years old with an overall age range of 18–50 years. Participants self-identified as 74% female, 21% male, and 5% transgender. The racial/ethnic makeup of our participants was: White (43%), Hispanic/Latino (23%), Black (23%), Biracial/multiracial (8%), and Asian American (3%). Participants described themselves as: older sisters (53%), older brothers (13%), aunts (13%), uncles (8%), cousins (8%), and siblings (5%, two non-gender binary participants). Close to half of the participants had completed some college (49%). Forty-six percent of participants completed college and 5% had a high school degree or less. All participants reported talking with the teens in their families about sex or relationships, with 85% of participants reporting that they talk with the teens “sometimes” or “often”.

### 2.2. Interview Protocols and Procedures

To ensure their privacy, participants were asked to create pseudonyms that would be used in this article and other publications. With participants’ permission, interviews were conducted over the phone, recorded, and later transcribed. In order to obtain accurate transcription, a transcriber with many years of experience transcribed the interviews and noted areas where recordings were unclear. In these cases, the researchers went back to the original recordings to clarify the language. Interviews took 45 min to one hour. Participants were reminded at the start of their interviews that their responses would be kept confidential. Participants were told that it was normal to feel embarrassed or uncomfortable talking about sexual topics and that they could stop the interview at any time. The interview protocol began by asking participants to describe their relationship with the teen with whom they talk about sex and relationships. Participants were asked how often they talk to this teen and what messages/topics they discuss when talking about sex or relationships.

Coding for this paper focused on four key areas related to the different roles of family members in talk with teens about sex or relationships. First, interviews addressed why teens talk with extended family participants about sex or relationships, “Why do you think [teen’s name] talks to you about these issues?” Next, participants were asked whether and how different family members talk with the teen about sex or relationships, including parents and extended family, e.g., “Do you know if [teen’s name] talks with other family members about sex or relationships? Why does [teen’s name] talk/not talk with them?” Interviews also addressed the consistency of messages across family members, “Would you give an example of a time when you and other family members were on the same page (or not) with the advice you gave [teen’s name] about sex or relationships?” Finally, interviews addressed whether and how family members communicated about the teen’s relationships or sexual behavior, “Could you think of a time that you and other family members talked with each other about [teen’s name] relationships? [If yes] How did the conversation go?”

Participants were also asked about their age, gender, racial/ethnic background and culture, and their education. Once an interview was completed, all participants were sent an e-mail that included a list of resources and contact information for organizations that support teens’ social, emotional, and sexual health. The $25 gift card to Target, CVS, or Amazon was sent via e-mail to the participant after the interview.

### 2.3. Data Analysis

Interview transcripts were coded using thematic analysis [36] to analyze for cross-cutting themes regarding the roles of the participant and other family members in talking with the teen about sex or relationships, differences and similarities in these conversations, and whether and how the participant and other family members talked with each other about the teen’s relationships or sexual health. The first and second authors summarized their preliminary reflections on family processes related to sexuality communication for each interview and, together, identified initial descriptive and interpretive codes. Next, they coded the interview data, and reviewed, revised, and defined themes. Themes are not mutually exclusive, because interview responses could be coded for more than one theme. After primary identification of themes, the first and second authors discussed their understanding of each code and revised coding definitions as needed. They reviewed one another’s coding by conducting reliability checks to synthesize cohesive subthemes and takeaways from each interview, resolving any discrepancies or inconsistencies between individual analyses. To protect against investigator bias and ensure they understood themes in similar ways, they calculated inter-rater reliability using Miles and Huberman’s formula [37], in which reliability equals the number of agreements divided by the total of agreements and disagreements. After several rounds of coding and discussion of disagreements, they reached a high level of intercoder reliability (96%) between the two coders. Coding and analysis used NVivo 10.0, manufactured by QSR International in Melbourne, Australia [38].

The authors calculated the percent for each theme based on the number of participants who were coded with a certain theme out of the total number of participants and for subthemes, percentages reflect the number of participants who were coded with a certain subtheme out of the total number of participants who provided information on that theme.

## 3. Results

The authors identified four themes regarding the roles of the participant and other family members in talking with the teen about sex or relationships. The first theme, *Why Teens Talk with Extended Family about Sex or Relationships*, focuses on participants’ perceptions of teens’ motivations for talking with them about sex or relationships. The second theme, *Family Engagement in Talk with Teens about Sex or Relationships*, addresses whether and how family members, including parents and extended family, talk with teens in the family about sex or relationships. The third theme, *Consistency of Messages to Teen across Family Members*, addresses family members’ similar and conflictual messages to teens about sex or relationships. The final theme, *Communication between Family Members about Teen Sex or Relationships*, relates to how family members talk with one another (or do not talk) about sex or relationships in relation to the teen. See Table 1 for the percentages of extended family members who discussed each theme and subtheme.

### 3.1. Why Teens Talk with Extended Family about Sex or Relationships

The first theme looks at participants’ perceptions of what motivates the teens to talk with them about sex or relationships. It explores the conditions and/or characteristics that facilitate teen–extended family communication. Almost all participants addressed this theme (97%). Four main subthemes emerged within this code: Connection with participant (92%), Discomfort with or lack of access to other family (63%), Shared demographics (50%) such as age or gender, and Shared personality or experience (39%).

The subtheme, Connection with participant, describes qualities of teens’ relationships with extended family, such as closeness and trust, which promote talk about sex or relationships. Many participants described how teens in their families trusted them with questions, private information, or concerns related to sex or relationships. For example, Ava described her nephew’s trust in her to keep their talk about sex or relationships between them, “Our conversations stay between us, so I think there’s like that level of trust there”. Alternatively, Jennifer described her close and comfortable relationship with her cousin, “I asked her, ‘Do you talk about this to your brothers or your sisters?’ She’s like ‘No’., She doesn’t feel comfortable telling them anything. And she feels more safe and comfortable with me. Because we just have that connection”.

The subtheme, Discomfort with or lack of access to other family, captures ways that teens turn to extended family for talk about sex because of a lack of connection to or availability of parents or other family. Participants describe some family members as unreceptive, uninterested, or unwilling to engage in the conversation. Jebin described how her brother was not comfortable talking about sex and relationships with their parents because “our family is very religious and like we’re not like really that open to our parents about like sex like or dating”. In this case, Jebin believed that her brother did not have access to their parents, since their religious beliefs precluded talk with them about sex and relationships. Similarly, Peter discussed why his brother doesn’t talk with his parents about sex, “I think there’s just kind of a general—in my family, a mentality of, you know, you shouldn’t ask things like that. You shouldn’t ask those questions”.

The subtheme Shared demographics, addressed instances where the participant specifically talked about age, gender, or sexuality as reasons why the teen talks to them about sex or relationships. About half of the responses within this subtheme mentioned similar age and same gender as factors that facilitate talk with the teen in their family about sex and relationships. Participants perceived these shared characteristics as a bridge that supported talk about sex or relationships. For example, when asked why her teenage cousin talks to her about sex or relationships, Anna K shared, “I think it’s definitely nice to have like a girl close to her age but a little older that will talk with her about these kinds of things that maybe she would feel uncomfortable telling her dad”. Rachel talked about how her shared sexual orientation with her sister made it easier for them to talk about sex, “I think it’s partly that we’re both queer. So like—you know, I’m not going to be just like thinking about like teen pregnancy because like I recognize that’s not super relevant to either of us. And I’m not going to be judgmental and like I’m, you know, going to like know what queer sex is um and things like that”.

The subtheme, Shared personality or experience, highlights the importance of sharing commonalities in facilitating a conversation about sex or relationships between teens and extended family members. Participants who talked about shared personalities often described similar interests, anxieties, social circles, and temperaments. Participants also talked about common experiences with relationships and sex making it easier to talk about these topics with the teen. For example, Jenny Reyes talked about how shared personality with her brother makes it easy for them to talk about sex and relationships, “I believe that he comes to me, again, because of the personality similarities that we have. Like he feels that he’ll be able to get the response he wants. And, you know, ever since we were little, I’ve been the—like the sister that he always goes to when he’s scared or he wants to feel, you know, safe”.

With regards to sharing similar experiences, Megan describes herself as one of her niece’s go-to people to talk about her relationship. “The same thing happened to me. I was in high school, the whole with the guy forever type of thing, and actually married the guy and it turned out horribly”, she laughs, “I don’t know if she’s almost worried the same thing might happen?” In this exchange, Megan shows that having a similar lived experience to her niece’s current relationship makes her a relevant and reliable outlet for her niece to bring any questions or concerns about sex or relationships.

### 3.2. Family Engagement in Talk about Sex or Relationships

The second theme addresses whether and how family members, including parents and extended family, talk with teens in the family about sex and relationships. Almost all participants addressed this theme (97%). Family engagement was coded regardless of whether the communication was health-promoting or how it was received by the teen. This theme included three subthemes: Mixed engagement (53%), All engaged (37%), and Only participant engaged (11%).

The first subtheme, Mixed engagement, reflects situations where some family members talk with the teen about sex or relationships, and others do not. Participants varied in their patterns of reported engagement, such as engagement from multiple extended family members but not parents, and engagement from a mother and sibling but not a father. For example, Tina talked about how her younger brother talks with some siblings, but not others, about sex, “Both me and my younger sister, who’s just a year younger than me, the two of us are very open about talking about relationships and sexuality and things like that. So especially when the three of us are hanging out, it comes up fairly often as a topic. I feel like he doesn’t talk to my older siblings—and I said that because I don’t think they talk very often in general. It’s more of a proximity and convenience question than, you know, a specific kind of discomfort”.

In other families, religious or cultural issues prevent some family members from talking with teens about sexual issues. Amy described variation in whether family members talk with her sister about sex or relationships, “I know she does have like an aunt that’s like really open and stuff, but I don’t really know like—I just know that her mom is—is really religious and doesn’t like to talk about that stuff, like is really against the LGBTQ community”.

The second subtheme, All engaged, focuses on families where the participant describes all family members as talking with the teen about sex or relationships. In many cases, multiple family members talk about sex or relationships in ways that support the teen’s health. For example, Mary described ways she and other family members talk with her younger brother, “My family, specifically my mother, my sister, and I have worked very hard to foster the sense of like openness, specifically in terms of sexuality and experiences and relationships … My dad has [also] brought some very valuable experiences to the conversation. Like he has mentioned how, you know, when my dad was like 19 he had a girlfriend and he ended up getting her pregnant”.

Other participants described multiple relatives engaged in talk about sex and relationships, but some of this engagement is more conflicted or contrasts with the teen’s beliefs and identity. For example, Gerald described the dynamics with his niece and her mother, “Usually it’s her talking to her mom and getting upset with the reaction from her mom. And then she’ll come—she’ll come to me and ask for my advice on it … .So she’s like, ‘I introduced my girlfriend to my mom and my mom got mad and said, “Why do you have a girlfriend? You’re a girl”‘. And she didn’t like that and she came to me and she was like, ‘Is it wrong?’ And I’m like, ‘No, it’s not wrong. It’s who you are’”.

The subtheme, Only participant is engaged, describes families in which the extended family participant is the only family member talking with the teen about sex or relationships, due to issues such as geographic distance, lack of closeness, and discomfort talking about sex or relationships. Sara explained why her sister doesn’t talk with their father about sex or relationships, “She feels very anxious and like mistrustful of our dad … I don’t think she talks to him at all”. Other participants described how some family members were not available to talk with them about sex, such as how Alvin described his cousin’s situation, “His mom is focused on his sisters…she’s a single mom essentially. So I don’t think there’s a lot of time for that”.

### 3.3. Consistency of Messages across Family Members

The third theme addresses whether and how family members, including parents and other relatives, shared similar or conflictual messages with teens about sex or relationships. Thirty-one (79%) participants addressed this issue. Subthemes include Similar messages (42%), when multiple family members shared consistent messages with the teen. Mixed messages (32%) was coded when participants described some family members who shared similar messages (e.g., aunt, sister), while others shared conflicting messages (e.g., brother) or when family members shared similar messages with the teen about one topic (e.g., what is a heathy relationship) but conflicting messages about another (e.g., when it is acceptable to have sex). Finally, Conflicting messages (26%) was coded when family members shared contrasting messages with the teen.

The first subtheme, Similar messages, includes instances in which family members share compatible messages with the teen about sex or relationships. In many cases, multiple families shared messages about sex or relationships in ways that supported the teen’s health. For example, Jennifer talked about a shared family message to her cousin, “One of my cousins, we were talking about how she wants to lose her virginity and stuff like that. And she was only like 16. And then me and my other cousin were like, ‘No, you don’t want to do that. You want to wait’”. Maria also described similar messages that her parents and siblings share with her brother, “My parents are very open with me. So in turn we’re very open with (my brother), even though he doesn’t want to hear it … ‘if you do choose to have sex, just make sure that it’s someone who you actually care about and … you’ve known and you’ve gotten tested and all that stuff’. And so we kind of say the same thing to him”.

The second subtheme, Mixed messages, includes instances when participants described both similar and conflicting messages family members shared with the teen about sex or relationships. This subtheme primarily addresses variation across family members (e.g., participant and teen’s grandmother give similar messages, while teen’s brother gives different messages), but also includes inconsistent messages shared by the same family members (e.g., participant and teen’s mother give similar messages about dating, but conflicting messages about sex). For example, Lucy contrasts her mother’s messages to her brother with the messages Lucy and her sisters share with him, “She’s (teen’s mother) just sort of like, ‘I want him to focus on school, that’s it. That’s all I want him to do. And I feel like if you guys are buying the condoms, you are promoting for him to be sexual’. And it’s not like that. We (participant and her sisters) know what’s going to happen, so we’re just making sure that he’s safe”.

In another case, Peter described how family members communicate similar messages to his brother about relationships, “Everyone (in the family) was kind of on the same page of, you know, if you’re not happy, if you don’t feel that kind of respect for the relationship or in the relationship, um you know, you’re not obligated to stay in it”. In contrast, Peter’s family members share different messages with his brother about whether it’s okay to talk about sex, “I think there’s a way it’s been a bit of a taboo around the topic of sex in my family … I never really subscribed to the taboo very much. And though I never said that out loud, I think he (my brother) kind of understood that”.

The third subtheme, Conflicting messages, includes instances in which family members share contrasting messages with the teen about sex or relationships, often related to whether and when to delay sex. Talk about different topics related to sex or relationships would not meet the criteria for this subtheme. For example, Meghan talked about how she tries to counter her nephew’s brother’s messages to him about condoms, “Unfortunately his older brother is super anti-condom for whatever reason. So he (her nephew) keeps hearing that a lot—that they’re not comfortable, that it doesn’t feel good. So every time he hears that, I swoop in and I’m like, ‘But there’s all these varieties and you can try different ones to find what does feel good’”. Alex described how his own messages to his sister were different than those from her parents, “I know my parents told her more strict like, ‘Hey, don’t get pregnant’. But I’m like, ‘Hey, be safe by using these methods’”.

### 3.4. Communication between Family Members about Teen Sex or Relationships

The final theme focuses on how family members talk with one another (or do not talk) about sex or relationships in relation to the teen; this theme was addressed by 97% of participants. The subtheme Communication (71%) was coded any time there was talk between two or more family members about the teen’s sexual health or relationships. The subtheme Coordination (50%) represents a sub-set of families that fit within the Communication subtheme. In these families, participants described a more organized level of talk, in which family members actively worked together to plan support for or communication with the teen related to sex or relationships. The No communication subtheme (29%) was coded when there was no communication among family members about the teen related to sex or relationships.

The Communication subtheme was coded when participants described talk with other family members (not including the teen) about the teen, related to sex or relationships. Communication consists of sharing and exchanging information, such as giving family members updates about teen sex or relationships. Charlotte described an instance where her parents asked her about her brother’s behavior, “I think the only time I’ve talked to my parents about his (her brother’s) like romantic life is when he broke up with that girl like at Christmastime”, starts Charlotte, “He was really down and they had no idea why he was down um and why he was so moody. And they were like, you know, ‘Why?’ And I was like, ‘Oh, well there was this girl situation, so maybe that’s why.’ And then my mom was like, ‘Oh, that makes perfect sense’”.

Communication was primarily positive, but also could include negative interactions (such as two family members arguing about the teen’s sexual behavior). One example shows how some family members talk about ways they disagree about the teen’s relationships. Kathleen described her conversations with her mother about her younger brother, “I have had many debates (with my mother) in the past, but I think that like at some point you just sort of have to agree to disagree. But my mom’s like pretty open to having those conversations, even if she knows that like ultimately we’re not going to agree. So like I’ve certainly said things to the effect of like, you know, ‘I hope [my brother] is happy with like whatever kinds of relationships he chooses to have’. Whereas I think my mom has a little bit more of a narrow vision of what he should be doing”.

The subtheme Coordination represents a higher level of engagement between the extended family participant and other family members, where the family network not only communicates about teen sex or relationships, but also mobilizes to carry out a goal or specific action related to the teen. For example, Maria found it challenging to gauge if her nephew was sexually active or not because he did not want to answer her questions about sex, “When I saw him kind of close off like that, I then approached his dad later on and said, ‘Hey, can you make sure you talk to him about this, because I didn’t get an answer from him’”. Several participants described requests from a teen’s parent to talk with the teen about sex or relationships. Anna J shared, “So I actually started talking to [my cousin] when she was like twelve, she was in the softball league. And she had a crush on a boy and that was the first time she had a crush on a boy. She told her dad. He was like freaking out. So her dad is my uncle; he’s my mom’s brother. And so he calls me and he’s like, ‘You need to talk to her about boys, because she has a crush on someone’. And so that was kind of the initiation of like, ‘Oh, this is your role of—this is your role now’”.

The subtheme, No communication, includes participants who shared that they do not talk with other family members about the teen’s experiences with sex or relationship. Reasons for no communication include not having access to other family members and family members not being interested in or wanting to have conversations about these topics. Anna K described how she does not talk to her uncle (the teen’s father) about teen sex and relationships largely due to their lack of physical proximity, but encourages her cousin to talk with her father. “I mean I have not talked to him (the teen’s father) about this, you know?” shares Anna K, “I’ll frequently be like, ‘Well, does your dad know?’ That’s like my catchphrase: ‘Does your dad know?’” In this case, even if there is no communication between Anna K and her uncle, she still tries to promote communication between her teen cousin and the cousin’s parent.

On the other hand, Trish describes how her family’s culture gets in the way of talk with her parents about her younger sister’s experiences with sex or relationships. When asked about whether Trish collaborates with her parents around these topics, Trish responded, “My parents definitely don’t. Like I’m 28, my parents haven’t even had a conversation with me about that. We just don’t do it. Yeah. They’re—we’re Latino. We’re Caribbean. It’s like we don’t have those conversations. It’s pretty much don’t ask, don’t tell”.

### 3.5. Comparison of Themes for Sibling versus Non-Sibling Participants

Initial comparisons (Table 2) suggest that siblings may be more likely to describe Shared demographics and Shared personality or experience than non-sibling participants. For example, Jane described why her brother talks with her and her siblings about sex and relationships, “It’s really nice to have siblings to talk to about it who are like closer in age to you and get it and like—and can give you advice that I think is harder to hear from parents”. Siblings were also more likely to report that everyone in the family is engaged in sexuality communication (All engaged) compared to Mixed engagement of non-sibling participants. For instance, Jamie described how everyone in her family talks with her brother about relationships, “me and my parents both talk to him, saying, you know, ‘It’s not healthy to scream at someone. If you love somebody, you shouldn’t be having shouting matches regularly. You shouldn’t want to hit each other’”. In contrast, Jose described how his niece talks to her mother, but not her step-father, about sex, “She’s told me that she’s talked to her mother about that … but my sister’s husband—my brother-in-law, he doesn’t really bring anything into that. He just doesn’t want anything to do with it”.

Further, siblings are more likely to report that family members share Similar messages with the teen as compared to Conflicting messages, which were more often described by non-sibling participants. Charlotte described the messages she and her sister both share with their brother, “Something we have emphasized over and over again is like the female side of things. I think it’s like really important that he knows like how the female body works, that he knows that like this isn’t like just about your pleasure, it’s also about her pleasure and like her comfort level and stuff like that”. Non-siblings, like Aunt Shawn, were more likely to discuss conflicting family messages, “Apparently his mom had told him about the pull-out method. And I was like, ‘I think your mom was pulling out when she had your sister at 16, so you might not want to follow her advice’”.

## 4. Discussion

This study is unique in its exploration of how extended family, parents and adolescents interact and shape the adolescents’ family system of sexual socialization. Though most research about conversations about sex and relationships with teens focuses on the parent–teen dyad, this study explores the roles that extended family members can play in the larger family system of sexuality communication. This study investigates extended family members’ perceptions of why teens talk with them about sex and relationships, whether and how family members talk with teens about sexual issues, whether messages to teens are consistent across family members, and whether and how family members talk to each other and coordinate with each other about teens’ sexual health and relationships. This study’s findings identify variation in family engagement, communication, and messaging to teens about sex, suggesting the need to understand and address the multiple sources and approaches to family talk about sex and relationships.

The theme *Why Teens Talk with Extended Family about Sex or Relationships* reflects the quality of teens’ relationships with extended family and parents as well as shared aspects of identity, experiences, and characteristics between teens and extended family members. Extended family participants’ talked about closeness and commonalities in experiences and characteristics as reasons for talk with adolescents in their families about sex, echoing previous research with adolescents’ who describe connections and shared life experiences as primary reasons they talk with extended family members about sex [5,9]. Teens may see extended family feedback about sex as highly relevant due to these similarities. The fit of the current findings with research on teens’ perspectives suggests that extended family members and adolescents view their reasons for talk about sex similarly, unlike findings that adolescents and their parents may not see eye to eye regarding their talk with one another about sex and often disagree about whether they have talked to each other about sexual topics [39,40]. Participants also described trust as a reason why teens talk with them about sex and relationships. Teens themselves also identify trust as a reason for talking with parents and extended family about sex, with a focus on seeing them as guides and believing they will be honest with them [5].

The theme *Family Engagement in Talk with Teens about Sex or Relationships* provides a window into the range of family member engagement in talk with teens about sex and relationships. Half of the participants described variation in whether their family members talked about sexual issues with teens, while other participants described their families as fully engaged in talk about sex, or only the participant talking with the teen. These findings suggest that family roles in talk about sex are not monolithic and that there are multiple configurations in families where the extended family are involved in talk with teens about sex. These results are consistent with quantitative findings that identified a range of adolescent reports about whom they talk to about sex: 21% of adolescents talk with parents only about sex, 18% talk with extended family only, 41% talk to both parents and extended family, and 21% talk to neither parents nor extended family [9].

The theme, *Consistency of Messages across Family Members*, adds another layer to our understanding of variation in family systems of talk with teens about sex and relationships. Even if all family members talk with the teen about sex or relationships, their messages may not be consistent. Our findings suggest that less than half of families share similar messages with teens, while others may pass on conflicting messages. Although prior studies have not directly explored this topic, teens’ reports suggest both similarities and differences in messages about sex or relationships from parents and extended family, with parents more likely to talk with teens about delaying sex and avoiding teen pregnancy, but similar likelihood of talk about using protection, being careful in relationships, and avoiding peer pressure [5]. Our results also suggest that family talk with teens about sex or relationships is not inherently positive or accurate. While some families provide consistent and health-promoting messages, most families provide a more mixed set of messages to teens. This complicates teens’ experiences of family talk about sex, as they may need to assess multiple messages to determine which messages are accurate or fit their beliefs and identities. This inconsistency of messages also complicates sex education efforts to increase teens’ talk with parents and other family members about sex, emphasizing the need for family support to engage in accurate, health-promoting communication with teens about sex and relationships.

Finally, the theme *Communication between Family Members about Teen Sex or Relationships* addresses whether and how family members talk with each other about teens’ sexual health and relationships. This theme gives another indication of how families navigate talk with teens about sex and relationships. To our knowledge, no other studies assess how parents and extended family interact with each other regarding teens’ sexuality communication and sexual health. As with the other themes, this data highlights variation in the extent to which family members collaborate to support teens’ sexual health and relationships. Half of families reach a high level of communication, in which they work together to support teens’ health, while many involve less communication or do not communicate at all.

An initial comparison of sibling and non-sibling participants in this study suggests both similarities and differences in how these groups engaged with teens in their families about sex and relationships. While both groups believed that close relationships were key to why teens talk with them about sex and relationships, siblings appeared more likely to focus on commonalities in background (e.g., shared gender, age), or experiences (having gone through a similar relationship). This fits with prior research that siblings relationships are partially consistent with parent–child roles. while also resembling peer connections [34,41]. Sibling participants also seemed more likely to describe working as a team with other family members, particularly parents and other siblings, who also engaged in talk about sex and shared similar messages with teens. In contrast, non-sibling participants were more likely to report mixed family engagement and conflicting messages. These findings suggest a potentially different role for non-sibling family, who may be more likely to fill in for parents or address parental messages that conflict with a teen’s values or identity. Given the lack of research on sibling and non-sibling roles in talking with teens about sex and relationships, future studies are needed to better understand their roles in larger family systems of sexuality communication and how sibling and non-sibling roles are similarly different.

This study begins to explore the role of family systems in talk with teens about sex and relationships. The findings provide initial support that competing family systems theories of congruence and compensation both may apply to family sexuality communication. Congruence approaches [23] fit with our findings that siblings and parents are often highly engaged and collaborative to support teens’ sexual health. In contrast, compensation approaches [24] fit with our findings that some extended family members take on primary roles in sexuality communication with a teen in their family. This often occurs when parents are unavailable to talk with teens or have conflicting sexual values, beliefs or identities, consistent with findings that extended family relationships, such as grandparents, aunts, and uncles, may help support youth when parents are not accessible [21,42] Variation in roles for extended family is a key finding of this study. Participants who described conflicting messages or lack of family engagement or communication about sex often referred to religious or cultural taboos, preventing some family members from talking about sex or led to negative messages, such as LGBTQ identities. This fits with prior research, which identifies cultural and religious taboos related to family communication about sex and relationships, e.g., [27,31]. More research is needed to address how family systems theory can be used to understand family talk about sex and relationships, including the impact of communication with multiple family members on teens’ health and development and the role of cultural and/or religious beliefs in shaping family systems of talk about sex.

This study is limited by its convenience sample, which disproportionately includes individuals who are actively engaged in talking with teens in their families about sex and relationships. However, given the lack of studies that investigate intersecting parent and extended family roles in talk with teens about sex, these findings provide a much-needed start to understanding this issue. Future research would benefit from including multiple perspectives on family systems of sexuality communication, to identify similarities and differences in parent, teen, and extended family perceptions of family communication. Further, given the variation in family roles in talk with teens about sex, quantitative research could help identify which combinations of family talk about sex protect teens from risky sexual behavior. This study’s findings for differences in how siblings and other extended family described family talk about sex also suggest the need for additional research focused on sibling roles in talk with teens about sex, which can add to initial research suggesting a potential protective role for talk with siblings about sex in promoting teens’ healthy sexual attitudes and behaviors [33,34]. The current sample size did not allow for statistical comparison between responses of sibling and non-sibling groups, a limitation that could be addressed in future studies. The lack of grandparents is also a limitation of our study sample, and future research could add to early studies in this area, which suggests that some grandparents are open to and involved in talk with their grandchildren about sex [8,43]. In addition, participants were not consistently asked whether they lived with the teen they talked with about sex and relationships, and therefore we were unable to explore whether and how living configurations shape extended family members’ talk with teens about sex or relationships.

While parents are often viewed as the primary sexuality educators for their children [3], these results indicate that not all parents are able or willing to take this role. According to participants in our study, while some parents actively and thoughtfully talk with teens about sex or relationships, others do not talk with their teens about these topics, or engage with teens in ways that participants view as problematic for teens’ identities or sexual health. This fits with findings from a review of common parental barriers that interfere with parents’ talk with teens about sex, which identifies lack of sexual health knowledge, discomfort with talk about sex, and perceptions of teens’ lack of readiness to talk about sex [44]. Prior research identifies a key role for extended family sexuality communication in supporting teen’s sexual health [9–11]. The current study suggests that extended family communication can range from complementing and supporting parents’ roles, to “filling in” for parents who do not have the comfort or capacity to talk with teens about sex or relationships, to pushing back against what extended family members sometimes perceive as incorrect, unresponsive, or un-empathic responses to teens. In contrast to the current focus on parents in sex education interventions [45], programs should recognize and address the diversity of how families talk (or do not talk) about sexual issues. While parents often provide important resources for their teens’ talk about sex, some teens may experience talk with parents as negative or problematic [5]. Therefore, educators should not assume that a parent is the only or most constructive family resource for teens’ talk about sex and relationships. The adolescents themselves may be a good place to start, as they can identify their own key family resources for talk about sex [11]. Programs can work with teens to help them to identify and draw on those resources to support their sexual health.

## 5. Conclusions

This is the first study to investigate extended family perspectives on family systems of talk with teens about sex and relationships. These findings underscore the importance of exploring larger family systems to understand family sexuality communication, rather than only parent–teen dyads. They also identify variation across families in engagement in talk, messages family members share with teens, and communication between family members. Our findings highlight that there is not just *one* role for extended family to play in conversations about sex or relationships. Instead, there is a need to move toward a framework that recognizes complex family systems of parent, teen, and extended family communication.

## Figures and Tables

**Table 1.** Sexuality communication themes (N = 39).

Theme	Frequency
**Why teens talk with extended family about sex or relationships**	**38 (97%)**
*Connection with participant*	35 (92%)
*Discomfort with or lack of access to other family*	24 (63%)
*Shared demographics*	19 (50%)
*Shared personality or experience*	15 (39%)
**Family engagement in talk with teens about sex or relationships**	**38 (97%)**
*Mixed engagement*	20 (53%)
*All engaged*	14 (37%)
*Only participant is engaged*	4 (11%)
**Consistency of messages to teens across family members**	**31 (79%)**
*Similar messages*	13 (42%)
*Mixed messages*	10 (32%)
*Conflicting messages*	8 (26%)
**Communication between family members about teen sex or relationships**	**38 (97%)**
*Communication*	27 (71%)
*Coordination*	19 (50%)
*No communication*	11 (29%)

Note: Themes above are in **bold**, subthemes are in *italics.*

**Table 2.** Sexuality communication themes for sibling (N = 25) and non-sibling (N = 14) participants.

Theme	Sibling %	Non-Sibling %
**Why teens talk with extended family about sex or relationships**		
*Connection with participant*	23 (92%)	12 (86%)
*Discomfort with or lack of access to other family*	14 (56%)	10 (71%)
*Shared demographics*	15 (60%)	4 (29%)
*Shared personality or experience*	12 (48%)	3 (21%)
**Family engagement in talk with teens about sex or relationships**		
*Mixed engagement*	9 (36%)	11 (79%)
*All engaged*	13 (52%)	1 (7%)
*Only participant is engaged*	3 (12%)	1 (7%)
**Consistency of messages to teen across family members**		
*Similar messages*	10 (40%)	3 (21%)
*Mixed messages*	6 (24%)	4 (29%)
*Conflicting messages*	6 (24%)	2 (14%)
**Communication between family members about teen sex or relationships**		
*Communication*	19 (76%)	8 (57%)
*Coordination*	12 (48%)	7 (50%)
*No communication*	6 (24%)	5 (36%)

Note: Themes above are in **bold**, subthemes are in *italics.*
